# A dual-validated machine learning model for predicting 3-year mortality in atypical pulmonary carcinoid, a rare neuroendocrine tumor

**DOI:** 10.3389/fendo.2026.1901646

**Published:** 2026-08-03

**Authors:** Yong Chen, Xiaoping Luo, Hao Zhang, Jia Zhou, Yonglin Yu, Guilan Yang, Juan Chen

**Affiliations:** 1 Ningxia Key Laboratory of Clinical and Pathogenic Microorganisms, Institute of Medical Science, General Hospital of Ningxia Medical University, Yinchuan, China; 2 Department of Respiratory and Critical Care Medicine, General Hospital of Ningxia Medical University, Yinchuan, China; 3 Department of Stomatology, The Affiliated Hospital of North Sichuan Medical College, Nanchong, China

**Keywords:** atypical pulmonary carcinoid, machine learning, neuroendocrine tumors, prognostic model, risk prediction

## Abstract

**Objectives:**

Atypical pulmonary carcinoid (AC) is a rare intermediate-grade neuroendocrine tumor with substantial clinical heterogeneity and an unpredictable prognosis. Accurate estimation of fixed-horizon mortality risk remains challenging because of its rarity and limited AC-specific prediction tools. This study aimed to develop and evaluate an interpretable model for estimating 3-year all-cause mortality in patients with AC.

**Methods:**

Clinical data for patients with AC diagnosed between 2000 and 2021 were retrospectively obtained from the Surveillance, Epidemiology, and End Results (SEER) database. Patients diagnosed during 2000–2018 (n=1, 301) were randomly divided into a training set (n=910) and an internal test set (n=391). Patients diagnosed during 2019–2021 (n=446) constituted a temporal validation cohort within the same registry, and 45 patients treated at the General Hospital of Ningxia Medical University during 2015–2024 were used for preliminary independent single-center evaluation. Demographic, clinicopathological, and treatment variables were considered. LASSO regression and the Boruta algorithm selected 11 predictors: Grade, N_stage, M_stage, Bone_metastasis, Brain_metastasis, Liver_metastasis, Marital_status, Radiation, Chemotherapy, Age, and Tumor_Size. Seven machine learning models were developed to evaluate predictive performance, including Logistic Regression (LR), Extreme Gradient Boosting (XGBoost), Light Gradient Boosting Machine (LightGBM), Gradient Boosting Decision Tree (GBDT), Support Vector Machine (SVM), K-Nearest Neighbors (KNN), and Adaptive Boosting (AdaBoost). The SHapley Additive exPlanations (SHAP) approach was used to interpret feature importance.

**Results:**

LR showed the most consistent held-out performance, with an area under the receiver operating characteristic curve of 0.802 (95% CI: 0.751–0.852) in the internal test set, 0.839 (95% CI: 0.796–0.882) in the temporal validation cohort, and 0.904 (95% CI: 0.808–1.000) in the single-center cohort. Boosting models showed larger declines from apparent training to held-out performance. Decision curve analysis (DCA) suggested potential net benefit across selected threshold probabilities. SHAP identified Age as the largest contributor to model predictions.

**Conclusion:**

The LR-based model showed consistent discrimination and interpretability in internal and same-registry temporal evaluation, with promising but imprecise results in the small single-center cohort. Geographic transportability remains unestablished. Independent multi-institutional validation with fully separated preprocessing is required before clinical use.

## 1 Introduction

Pulmonary neuroendocrine neoplasms (PNENs) are clinically and biologically heterogeneous thoracic malignancies, ranging from low-grade typical pulmonary carcinoid (TC) to highly aggressive subtypes such as small cell lung cancer (1). Atypical pulmonary carcinoid (AC) is a rare intermediate-grade PNEN, accounting for 10%–15% of pulmonary carcinoids (2). Histologically, AC is defined by 2–10 mitoses per 2 mm² or focal necrosis, whereas TC presents fewer than 2 mitoses per 2 mm² without necrosis (3). Recent studies have further demonstrated biological heterogeneity within AC, including distinct tumor-immune microenvironment profiles and prognostically relevant variation in mitotic count and Ki-67 labeling indices (4, 5). Long-term outcomes are generally less favorable than those of TC. In a retrospective study with 10 years of follow-up, the 10-year cancer-specific survival (CSS) and disease-free survival (DFS) rates for AC were 49.1% and 52.2%, respectively, compared with 86.8% and 92.6% for TC (6).

Patients with surgically managed AC continue to experience substantial long-term risks of recurrence, metastasis, and mortality. Reported 10-year survival after surgical management is approximately 59% (7), and a previous study of low-to-intermediate-grade neuroendocrine tumors reported a 3-year overall survival rate of 68.1% for AC (8). These findings underscore the marked heterogeneity of AC and the need for clinically useful risk stratification. The TNM staging system remains an important anatomical framework for lung cancer prognosis (9). Previous studies have developed prognostic nomograms and clinical management frameworks for pulmonary carcinoid populations, including AC-specific models (10, 11); however, these tools differ in target population, prediction horizon, endpoint, predictor set, and validation strategy. Evidence specifically addressing fixed-horizon 3-year all-cause mortality in an AC-specific population therefore remains limited.

Machine-learning methods have increasingly been applied to diagnosis, prognosis, and risk stratification in rare diseases and oncology (12–15). These methods can evaluate complex interactions and nonlinear relationships among routinely collected variables, but their performance depends on sample size, predictor structure, data quality, and control of overfitting. Regression-based and machine-learning approaches should therefore be regarded as complementary rather than inherently competing. Systematic comparison of models with different levels of complexity may help identify an approach that balances discrimination, calibration, stability, and interpretability for rare-disease prediction.

The present study systematically compared seven candidate classifiers for estimating 3-year all-cause mortality in an AC-specific population. Performance was evaluated in a held-out internal test set, a later temporal SEER cohort, and a preliminary independent single-center cohort. SHAP analysis was used to explain the relative contribution of the selected predictors, while supplementary analyses assessed overlap among metastasis-related variables and incremental discrimination beyond simpler clinical specifications.

## 2 Patients and methods

### 2.1 Patients

#### 2.1.1 Subjects

This retrospective prediction-model study used SEER registry data (16) and an independent hospital cohort. Patients with AC diagnosed during 2000-2018 (n=1, 301) were randomly divided into a training set (n=910) and an internal test set (n=391). Patients diagnosed in SEER during 2019-2021 (n=446) formed a temporal validation cohort within the same registry. A separate cohort of 45 patients diagnosed at the General Hospital of Ningxia Medical University during 2015–2024 provided preliminary independent single-center evaluation. For SEER, 3-year mortality status was derived from survival time and vital status. For the single-center cohort, survival status was ascertained from medical records and follow-up information. The single-center study was approved by the Ethics Committee of the General Hospital of Ningxia Medical University (Approval No. KYLL-2026-0771), with informed consent waived for anonymized retrospective records. No formal *a priori* sample-size calculation was performed because cohort size was fixed by the eligible cases available within the specified study periods; all eligible patients were included.

#### 2.1.2 Inclusion criteria and exclusion standards

**Figure 1:**
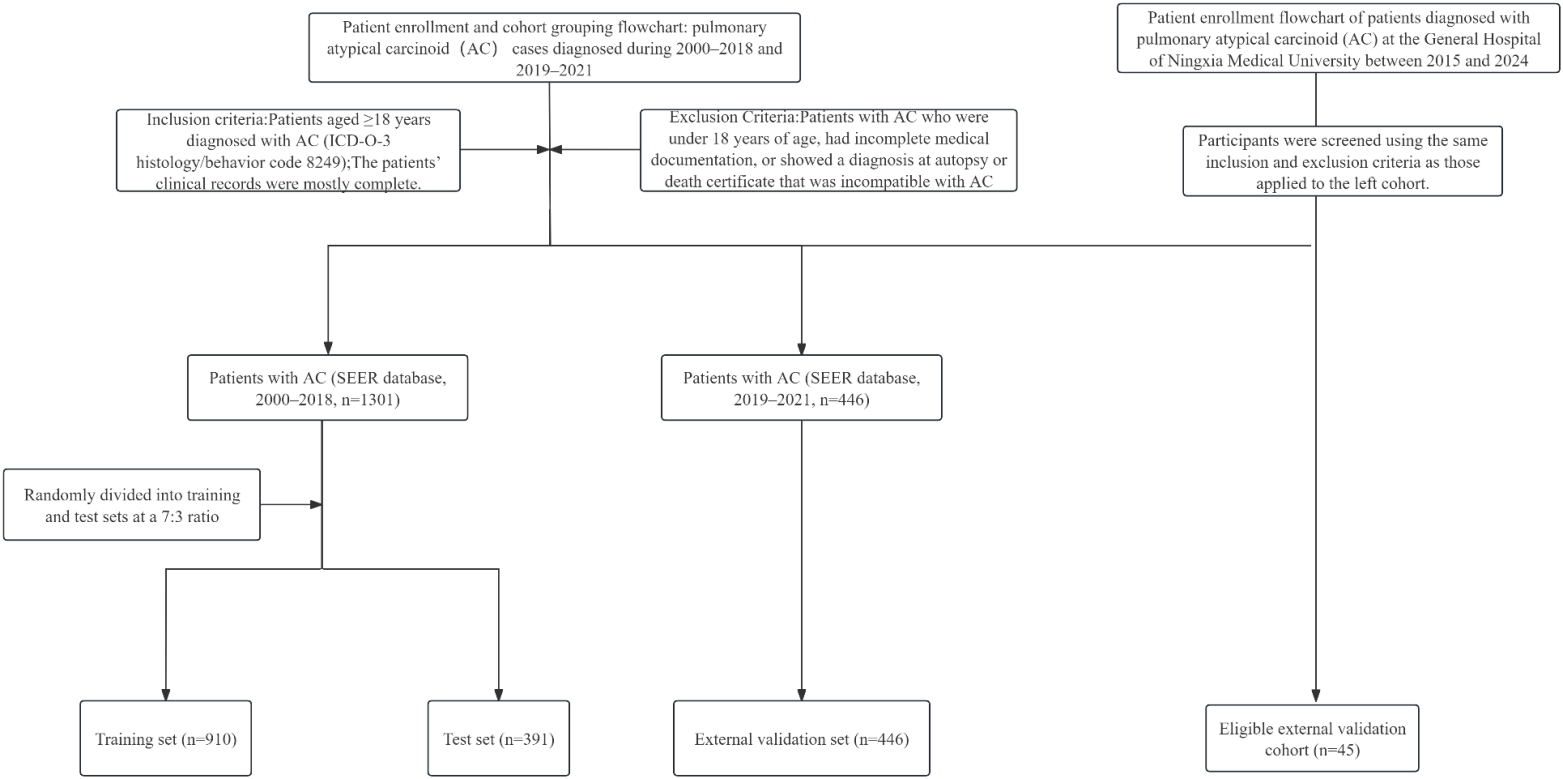
Participant flow diagram for the development and validation cohorts. Patients with atypical pulmonary carcinoid were identified in SEER for model development and internal testing (2000-2018) and temporal validation (2019-2021), and at the General Hospital of Ningxia Medical University for preliminary independent single-center evaluation (2015-2024).

Patients were eligible if they were aged ≥18 years and had atypical pulmonary carcinoid identified using ICD-O-3 histology/behavior code 8249. Patients diagnosed only by autopsy or death certificate or lacking the survival-time or vital-status information required to determine the 3-year outcome were excluded. Missing candidate-predictor values were not used as exclusion criteria and were addressed using multiple imputation. The single-center cohort was screened using corresponding eligibility criteria (**Figure 1**).

### 2.2 Methods

#### 2.2.1 Study indicators

The variables included in this study were as follows: Primary_Site, Grade, Laterality, T_stage, N_stage, M_stage, Systemic_Sur_Seq, Bone_metastasis, Brain_metastasis, Liver_metastasis, Lung_metastasis, Lymph_node_metastases, Marital_status, Sex, Race, Surgery_Radiation_Sequence, Radiation, Chemotherapy, Rural_Urban, Tumor_Size, and Age.

Outcome indicator: Survival_months and Vital_status.

The predicted outcome was all-cause death within 36 months after diagnosis. Death before 36 months was coded as an event, whereas survival to at least 36 months was coded as a non-event.

#### 2.2.2 Data preprocessing

Before model development, the number and percentage of missing observations were assessed for each candidate predictor in the complete SEER cohort of 1, 747 patients. Fourteen of the 21 candidate predictors contained missing values, with variable-specific missingness ranging from 2.7% to 52.6%. Missing data were handled using the Automatic multiple-imputation procedure in IBM SPSS Statistics version 25.0, with five imputations specified. For the non-monotone missingness pattern, fully conditional specification was used, with linear regression for scale variables and logistic-type regression models for categorical variables. The completed data file available from the original SPSS imputation workflow was exported for subsequent analyses in R and Python. Imputation was performed on the complete 2000–2021 SEER dataset before separation into the 2000–2018 development cohort and the 2019–2021 temporal validation cohort and before the subsequent 7:3 training-internal-test split. The final predictors were complete in the single-center cohort. This sequence did not preserve strict independence of preprocessing between the development and evaluation cohorts because information from later SEER cases contributed to the imputation models; its potential influence on optimism is addressed in the Limitations. The variable-specific missingness profile is provided in **Supplementary Table 3**.

#### 2.2.3 Model development and evaluation

Model development comprised the following steps. Candidate predictors were numerically encoded according to prespecified categories. The 2000–2018 development cohort was randomly divided into training and internal test sets at a 7:3 ratio. Feature selection using LASSO regression (17) and the Boruta algorithm (18) was then performed exclusively within the training set; the internal test set was not used for predictor selection or model fitting. Seven classifiers were compared: LR, XGBoost, LightGBM, GBDT, AdaBoost, SVM, and KNN. Ten-fold cross-validation was conducted within the training set, and the held-out internal test set was used for performance assessment. Discrimination was evaluated using ROC curves and AUC (19, 20), calibration was assessed using calibration plots, calibration intercepts, and calibration slopes, overall probabilistic accuracy was summarized using Brier scores, precision-recall performance was assessed (21), and clinical utility was explored using decision curve analysis (22). SHAP was used for *post hoc* interpretation of the selected model (23). The 2019–2021 SEER cohort was used to assess temporal performance within the same registry, whereas the hospital cohort was used for preliminary independent single-center evaluation. Available and verifiable implementation details are summarized in (**Supplementary Table 4**).

#### 2.2.4 Statistical analysis

Descriptive analyses were performed for all variables. Categorical variables are presented as counts and percentages and were compared using the chi-square test. Continuous variables were assessed for normality; non-normally distributed variables are presented as median and interquartile range and were compared using the Mann-Whitney U test. Model discrimination was summarized using AUC with 95% confidence intervals. Calibration was examined using calibration plots. Calibration intercepts and slopes were estimated separately in each evaluation cohort by fitting a logistic recalibration model of the observed outcome on the logit-transformed predicted probability; ideal values were 0 and 1, respectively, and Wald 95% confidence intervals were reported. Brier scores summarized overall probabilistic accuracy (21). Analyses were performed using SPSS version 25.0, R version 4.3.3, and Python version 3.11.9. All statistical tests were two-sided, with *P<0.05* considered statistically significant. The reporting of this study was guided by the applicable TRIPOD+AI recommendations for prediction-model studies (24).

To evaluate overlap among metastasis-related predictors, pairwise correlations and variance inflation factors were calculated. A *post hoc* sensitivity analysis compared the full 11-predictor specification with two reduced specifications: one excluding M_stage and one retaining M_stage while excluding Bone_metastasis, Brain_metastasis, and Liver_metastasis. A separate benchmark analysis compared the full specification with a TNM-only model and a parsimonious clinical model containing Age, Grade, Tumor_Size, T_stage, N_stage, and M_stage. All supplementary models were fitted using the same unpenalized logistic-regression procedure and evaluated by stratified 10-fold cross-validation in the 2000–2018 development cohort and temporal validation in the 2019–2021 SEER cohort. These comparisons were descriptive, were conducted specifically in response to the reviewers’ comments, and did not replace the primary analysis.

## 3 Results

### 3.1 Baseline characteristics analysis of AC patients with 3-year survival versus death

The median Age was 65 years (interquartile range, 55–72 years), and 432 patients (33.2%) were male. Patients who died within 3 years differed from those who survived for at least 3 years with respect to Primary_Site, Grade, T_stage, N_stage, M_stage, Bone_metastasis, Brain_metastasis, Liver_metastasis, Lung_metastasis, Lymph_node_metastases, Marital_status, Race, Radiation, Chemotherapy, Tumor_Size, and Age (all *P<0.05*). No statistically significant differences were observed for Laterality, Systemic_Sur_Seq, Sex, Surgery_Radiation_Sequence, or Rural_Urban (**Table 1**).

**Table 1:** Baseline characteristics: 3-year survival vs death in AC patients.

Variable	Overall (N = 1301)	Dead (N = 363)	Alive (N = 938)	*p*
Primary_Site, N (%)				0.007
Lower lobe	539(41.430)	138(38.017)	401(42.751)	
Main bronchus	69(5.304)	25(6.887)	44(4.691)	
Middle lobe	217(16.679)	46(12.672)	171(18.230)	
Overlapping	24(1.845)	6(1.653)	18(1.919)	
Upper lobe	452(34.743)	148(40.771)	304(32.409)	
Grade, N (%)				<0.001
GradeI	332(25.519)	66(18.182)	266(28.358)	
GradeII	861(66.180)	243(66.942)	618(65.885)	
Grade III	83(6.380)	40(11.019)	43(4.584)	
GradeIV	25(1.922)	14(3.857)	11(1.173)	
Laterality, N (%)				0.657
Left	500(38.432)	143(39.394)	357(38.060)	
Right	801(61.568)	220(60.606)	581(61.940)	
T_stage, N (%)				<0.001
T1	585(44.965)	127(34.986)	458(48.827)	
T2	382(29.362)	109(30.028)	273(29.104)	
T3	177(13.605)	63(17.355)	114(12.154)	
T4	157(12.068)	64(17.631)	93(9.915)	
N_stage, N (%)				<0.001
N0	755(58.032)	150(41.322)	605(64.499)	
N1	181(13.912)	50(13.774)	131(13.966)	
N2	314(24.135)	136(37.466)	178(18.977)	
N3	51(3.920)	27(7.438)	24(2.559)	
M_stage, N (%)				<0.001
M0	1023(78.632)	193(53.168)	830(88.486)	
M1	278(21.368)	170(46.832)	108(11.514)	
Systemic_Sur_Seq, N (%)				0.516
No	1117(85.857)	308(84.848)	809(86.247)	
Yes	184(14.143)	55(15.152)	129(13.753)	
Bone_metastasis, N (%)				<0.001
No	1216(93.467)	300(82.645)	916(97.655)	
Yes	85(6.533)	63(17.355)	22(2.345)	
Brain_metastasis, N (%)				<0.001
No	1260(96.849)	332(91.460)	928(98.934)	
Yes	41(3.151)	31(8.540)	10(1.066)	
Liver_metastasis, N (%)				<0.001
No	1170(89.931)	275(75.758)	895(95.416)	
Yes	131(10.069)	88(24.242)	43(4.584)	
Lung_metastasis, N (%)				<0.001
No	1223(94.005)	316(87.052)	907(96.695)	
Yes	78(5.995)	47(12.948)	31(3.305)	
Lymph_node_metastases, N (%)				<0.001
No	1255(96.464)	336(92.562)	919(97.974)	
Yes	46(3.536)	27(7.438)	19(2.026)	
Marital_status, N (%)				<0.001
Unmarried	213(16.372)	56(15.427)	157(16.738)	
Married	765(58.801)	188(51.791)	577(61.514)	
Divorced	136(10.453)	46(12.672)	90(9.595)	
Widowed	187(14.374)	73(20.110)	114(12.154)	
Sex, N (%)				0.848
Female	869(66.795)	241(66.391)	628(66.951)	
Male	432(33.205)	122(33.609)	310(33.049)	
Race, N (%)				0.025
Other	49(3.766)	15(4.132)	34(3.625)	
White	1144(87.932)	306(84.298)	838(89.339)	
Black	108(8.301)	42(11.570)	66(7.036)	
Surgery_Radiation_Sequence, N (%)				0.061
No radiation and/or no surgery	1192(91.622)	322(88.705)	870(92.751)	
Radiation after surgery	104(7.994)	39(10.744)	65(6.930)	
Radiation prior to surgery	5(0.384)	2(0.551)	3(0.320)	
Radiation, N (%)				<0.001
No	1112(85.473)	268(73.829)	844(89.979)	
Yes	189(14.527)	95(26.171)	94(10.021)	
Chemotherapy, N (%)				<0.001
No	1000(76.864)	225(61.983)	775(82.623)	
Yes	301(23.136)	138(38.017)	163(17.377)	
Rural_Urban, N (%)				0.21
Rural	159(12.221)	51(14.050)	108(11.514)	
Urban	1142(87.779)	312(85.950)	830(88.486)	
Tumor_Size, median [IQR]	24.000[16.000, 38.000]	29.000[19.000, 45.000]	23.000[15.000, 35.000]	<0.001
Age, median [IQR]	65.000[55.000, 72.000]	69.000[61.000, 77.000]	62.000[53.000, 71.000]	<0.001

Categorical variables are presented as n (%), continuous variables as median [Q1–Q3]. Q1, first quartile; Q3, third quartile. *P < 0.05* was considered statistically significant.

### 3.2 Comparison of baseline characteristics between the training and testing sets

The 2000–2018 development cohort comprised 1, 301 patients, of whom 910 were assigned to the training set and 391 to the internal test set. Baseline characteristics were generally comparable between the two sets, although rural–urban residence differed statistically *(P = 0.005*; **Table 2**).

**Table 2:** Baseline data were compared between the two datasets.

Variable	Overall (n=1301)	Training set (n=910)	Testing set (n=391)	*p*
Primary_Site, N (%)				0.38
Lower lobe	539(41.430)	393(43.187)	146(37.340)	
Main bronchus	69(5.304)	47(5.165)	22(5.627)	
Middle lobe	217(16.679)	148(16.264)	69(17.647)	
Overlapping	24(1.845)	15(1.648)	9(2.302)	
Upper lobe	452(34.743)	307(33.736)	145(37.084)	
Grade, N (%)				0.672
GradeI	332(25.519)	235(25.824)	97(24.808)	
GradeII	861(66.180)	604(66.374)	257(65.729)	
Grade III	83(6.380)	56(6.154)	27(6.905)	
GradeIV	25(1.922)	15(1.648)	10(2.558)	
Laterality, N (%)				0.099
Left	500(38.432)	363(39.890)	137(35.038)	
Right	801(61.568)	547(60.110)	254(64.962)	
T_stage, N (%)				0.323
T1	585(44.965)	398(43.736)	187(47.826)	
T2	382(29.362)	273(30.000)	109(27.877)	
T3	177(13.605)	132(14.505)	45(11.509)	
T4	157(12.068)	107(11.758)	50(12.788)	
N_stage, N (%)				0.275
N0	755(58.032)	532(58.462)	223(57.033)	
N1	181(13.912)	126(13.846)	55(14.066)	
N2	314(24.135)	211(23.187)	103(26.343)	
N3	51(3.920)	41(4.505)	10(2.558)	
M_stage, N (%)				0.511
M0	1023(78.632)	720(79.121)	303(77.494)	
M1	278(21.368)	190(20.879)	88(22.506)	
Systemic_Sur_Seq, N (%)				0.274
No	1117(85.857)	775(85.165)	342(87.468)	
Yes	184(14.143)	135(14.835)	49(12.532)	
Bone_metastasis, N (%)				0.911
No	1216(93.467)	851(93.516)	365(93.350)	
Yes	85(6.533)	59(6.484)	26(6.650)	
Brain_metastasis, N (%)				0.135
No	1260(96.849)	877(96.374)	383(97.954)	
Yes	41(3.151)	33(3.626)	8(2.046)	
Liver_metastasis, N (%)				0.743
No	1170(89.931)	820(90.110)	350(89.514)	
Yes	131(10.069)	90(9.890)	41(10.486)	
Lung_metastasis, N (%)				0.534
No	1223(94.005)	853(93.736)	370(94.629)	
Yes	78(5.995)	57(6.264)	21(5.371)	
Lymph_node_metastases, N (%)				0.55
No	1255(96.464)	876(96.264)	379(96.931)	
Yes	46(3.536)	34(3.736)	12(3.069)	
Marital_status, N (%)				0.079
Unmarried	213(16.372)	141(15.495)	72(18.414)	
Married	765(58.801)	556(61.099)	209(53.453)	
Divorced	136(10.453)	88(9.670)	48(12.276)	
Widowed	187(14.374)	125(13.736)	62(15.857)	
Sex, N (%)				0.623
Female	869(66.795)	604(66.374)	265(67.775)	
Male	432(33.205)	306(33.626)	126(32.225)	
Race, N (%)				0.472
Other	49(3.766)	35(3.846)	14(3.581)	
White	1144(87.932)	805(88.462)	339(86.701)	
Black	108(8.301)	70(7.692)	38(9.719)	
Surgery_Radiation_Sequence, N (%)				0.85
No radiation and/or no surgery	1192(91.622)	832(91.429)	360(92.072)	
Radiation after surgery	104(7.994)	74(8.132)	30(7.673)	
Radiation prior to surgery	5(0.384)	4(0.440)	1(0.256)	
Radiation, N (%)				0.891
No	1112(85.473)	777(85.385)	335(85.678)	
Yes	189(14.527)	133(14.615)	56(14.322)	
Chemotherapy, N (%)				0.62
No	1000(76.864)	696(76.484)	304(77.749)	
Yes	301(23.136)	214(23.516)	87(22.251)	
Vital_status, N (%)				0.599
Dead	363(27.902)	250(27.473)	113(28.900)	
Alive	938(72.098)	660(72.527)	278(71.100)	
Rural_Urban, N (%)				0.005
Rural	159(12.221)	96(10.549)	63(16.113)	
Urban	1142(87.779)	814(89.451)	328(83.887)	
Tumor_Size, median [IQR]	24.000[16.000, 38.000]	25.000[15.000, 38.000]	24.000[16.000, 40.000]	0.566
Age, median [IQR]	65.000[55.000, 72.000]	65.000[55.000, 72.000]	65.000[56.000, 73.000]	0.671

Categorical variables are presented as n (%), continuous variables as median [Q1–Q3]. Q1, first quartile; Q3, third quartile. *P < 0.05* was considered statistically significant.

### 3.3 Risk factors associated with 3-year mortality in AC

**Figure 2:**
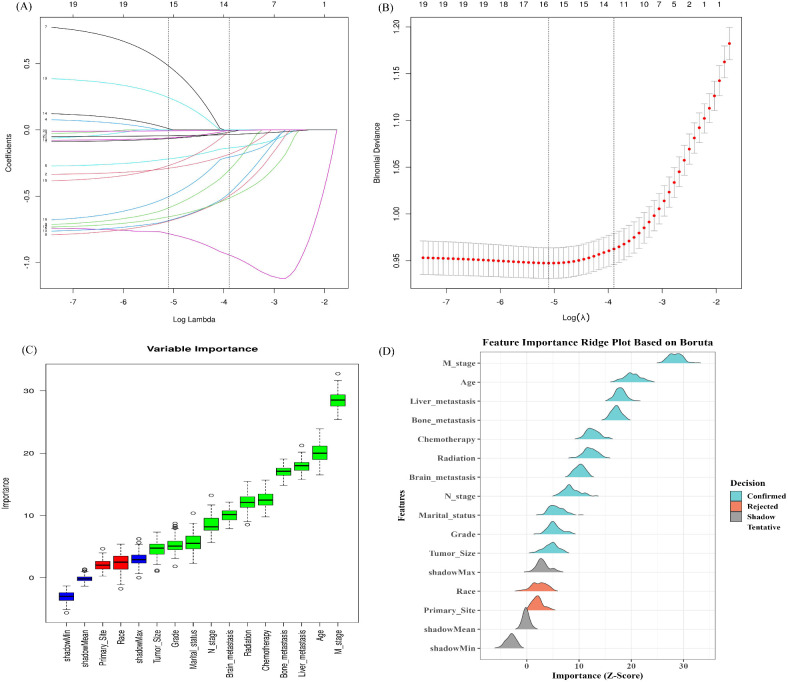
Feature selection for the prediction model using LASSO regression and the Boruta algorithm. **(A)** LASSO coefficient paths display how feature coefficients shrink with increasing regularization strength (log λ). **(B)** Ten-fold cross-validation selects the optimal λ by minimizing the mean cross-validated binomial deviance. Vertical dotted lines indicate λ_min (the value minimizing the mean cross-validated binomial deviance; λ = 0.006) and λ_1SE (the largest λ within one standard error of the minimum; λ = 0.02). **(C)** The Boruta box plot compares feature-importance scores with shadow variables, highlighting confirmed (green), tentative (blue), and rejected (red) features. **(D)** The Boruta ridge plot visualizes Z-score distributions, showing the importance distributions of the selected clinical predictors (cyan) relative to shadow variables (gray).

To identify a concise predictor set, LASSO regression was applied within the training set, with the penalty parameter selected using the lambda.min criterion (**Figures 2A, B**). Boruta analysis was then used to assess variable importance (**Figures 2C, D**). Within the Boruta importance output, M_stage ranked highest, followed by Age, Liver_metastasis, Bone_metastasis, Chemotherapy, Radiation, Brain_metastasis, N_stage, Marital_status, Grade, and Tumor_Size. The final 11 predictors were Grade, N_stage, M_stage, Bone_metastasis, Brain_metastasis, Liver_metastasis, Marital_status, Radiation, Chemotherapy, Age, and Tumor_Size. The Boruta ordering reflects feature-selection importance and is distinct from the final-model SHAP ranking. These variables were carried forward for model development.

### 3.4 A comprehensive multi-model analysis framework for classification

**Figure 3:**
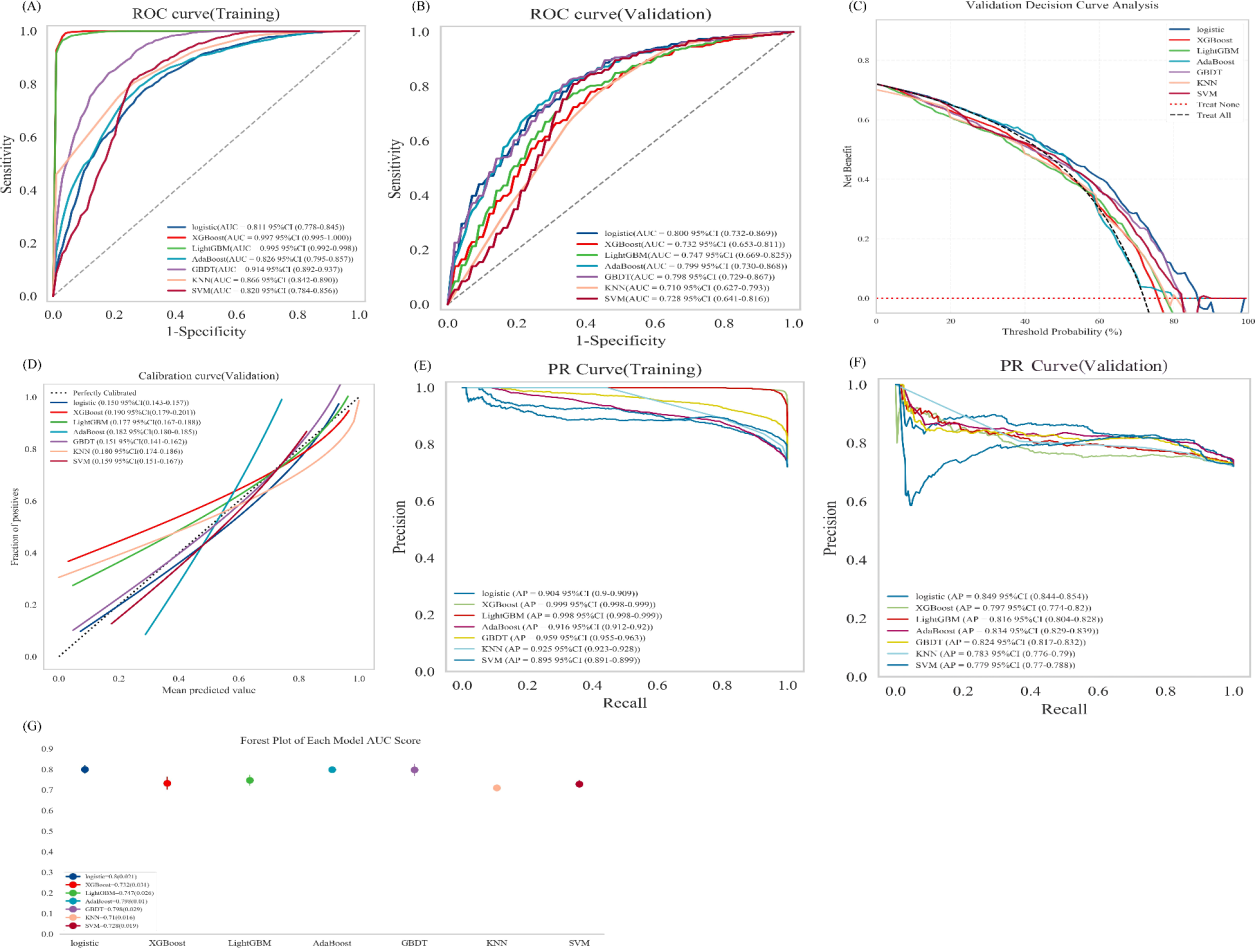
Comprehensive performance evaluation of the seven candidate classifiers. **(A, B)** ROC curves and corresponding AUC values in the training set and held-out internal test set, respectively. **(C)** Decision curves in the internal test set, with the dashed lines representing the treat-all and treat-none strategies. **(D)** Calibration curves for the candidate models. **(E, F)** Precision-recall curves in the training and internal test sets. **(G)** Forest plot summarizing model AUCs.

Across the candidate algorithms, LR showed the most consistent overall performance profile. In the initial seven-classifier comparison, its apparent training-set AUC was 0.811 (95% CI: 0.778–0.845), whereas its held-out internal-test AUC was 0.800 (95% CI: 0.732–0.869) (**Figures 3A, B**). In contrast, XGBoost and LightGBM achieved very high apparent training discrimination but substantially lower performance in the held-out data, a pattern consistent with overfitting. Decision curve analysis suggested potential net benefit across selected threshold probabilities (**Figure 3C**). The calibration plot showed visual agreement between predicted and observed risks, and the Brier score was 0.150 (95% CI: 0.143–0.157) (**Figure 3D**). In the precision–recall analysis, LR achieved a validation average precision of 0.849 (95% CI: 0.844–0.854) (**Figures 3E, F**). The forest plot summarized the AUC estimates across classifiers (**Figure 3G**). Taken together, these findings supported selection of LR as the final model while indicating that increased algorithmic complexity did not improve held-out performance in this dataset (**Supplementary Tables 1, 2**).

The final predictors had variance inflation factors ranging from 1.08 to 2.49. Correlations between M_stage and Bone_metastasis, Brain_metastasis, and Liver_metastasis were 0.507, 0.335, and 0.636, respectively. In the *post hoc* sensitivity analysis, the full predictor specification achieved AUCs of 0.787 in 10-fold cross-validation and 0.843 in temporal validation. The corresponding AUCs were 0.782 and 0.832 after excluding M_stage and 0.785 and 0.838 after retaining M_stage but excluding Bone_metastasis, Brain_metastasis, and Liver_metastasis. Performance was broadly maintained across the three specifications; the comparisons were descriptive and no formal hypothesis test of AUC differences was performed (**Supplementary Table 5**).

### 3.5 Optimized model construction and evaluation

**Figure 4:**
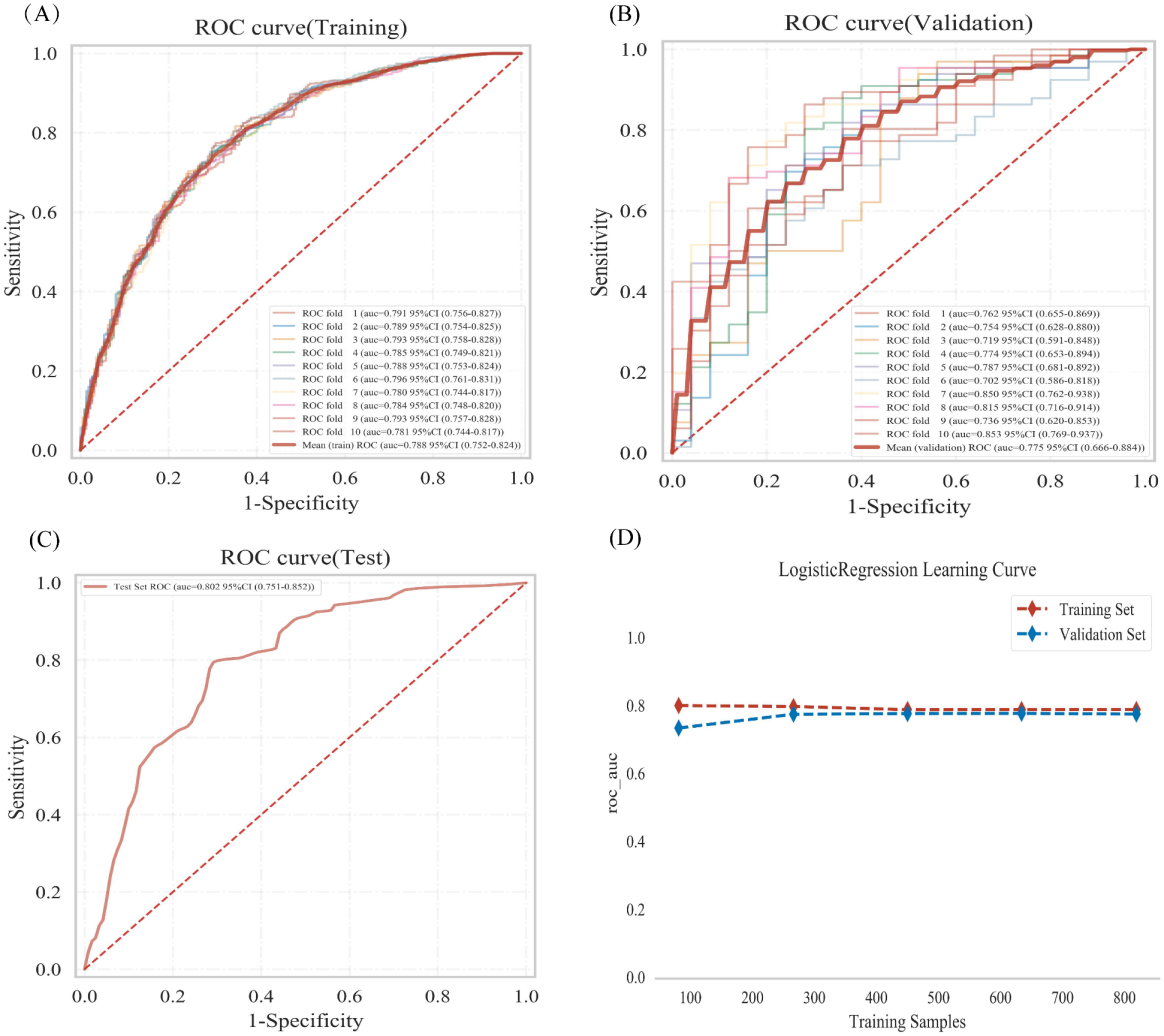
Internal evaluation of the selected LR model. **(A, B)** ROC curves for the training and validation folds from 10-fold cross-validation; thin lines represent individual folds and thick lines represent mean performance. **(C)** ROC curve and AUC in the held-out internal test set. **(D)** Learning curve showing training- and validation-fold performance as the training sample size increased.

After LR was selected in the seven-classifier comparison, the final LR model was evaluated using 10-fold cross-validation within the training set and then assessed in the held-out internal test set. The mean AUC was 0.788 (95% CI: 0.752–0.824) in the training folds and 0.775 (95% CI: 0.666–0.884) in the validation folds, while the held-out internal-test AUC was 0.802 (95% CI: 0.751–0.852) (**Figures 4A–C**). The small difference from the AUC of 0.800 in the initial seven-classifier comparison reflects separate evaluation outputs for classifier comparison and final-model assessment. The learning curves showed convergence between the training and validation results (**Figure 4D**). These findings support acceptable internal discrimination; however, the preprocessing sequence described in Section 2.2.2 may have introduced optimism.

In the *post hoc* benchmark analysis, the full 11-predictor specification, parsimonious clinical specification, and TNM-only specification achieved cross-validated AUCs of 0.787, 0.775, and 0.704, respectively. The corresponding temporal-validation AUCs were 0.843, 0.816, and 0.775. The full specification therefore showed numerically higher discrimination in this descriptive comparison. This benchmark was limited to internally constructed comparison models; no published nomogram or score was directly applied, and superiority over existing prognostic tools cannot be inferred (**Supplementary Table 6**).

### 3.6 Model interpretation using SHAP

**Figure 5:**
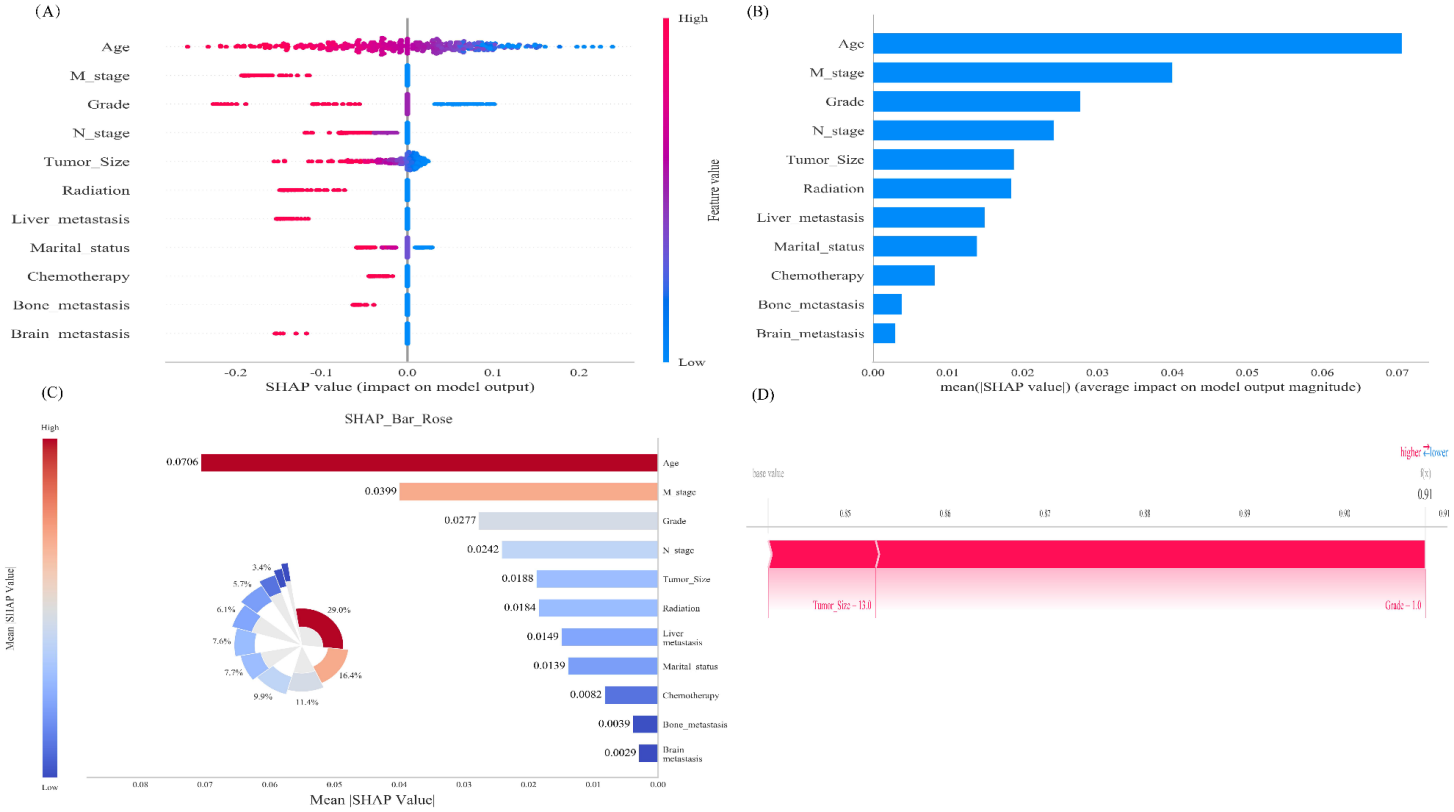
SHAP analysis for interpreting the AC prognostic model. **(A)** The SHAP summary dot plot shows the distribution of predictor contributions to model output. Each dot represents one patient and is colored according to the encoded feature value (pink = higher, blue = lower); positive and negative SHAP values indicate contributions toward higher and lower model output, respectively. Because several categorical predictors were numerically encoded, direction should be interpreted with reference to the coding scheme rather than as a simple biological gradient. **(B)** Mean absolute SHAP values rank the overall magnitude of each predictor’s contribution, with Age ranked first, followed by M_stage, Grade, N_stage, and Tumor_Size. **(C)** The bar and rose plots summarize the mean absolute SHAP values and proportional contributions (e.g., Age: 0.0706 and 29.0%; M_stage: 0.0399 and 16.4%). **(D)** The force plot illustrates how the encoded predictor values for one representative patient jointly shift the model output from its baseline value. This panel demonstrates individual-level model explanation and should not be interpreted as evidence of causal effects.

SHAP analysis was used to interpret the relative contribution of the predictors and their direction with respect to the fitted model output rather than to claim discovery of previously unknown biological factors. Because several categorical predictors were numerically encoded, directional interpretation requires reference to the coding scheme and model-output class. The SHAP summary plot showed the distribution of each predictor’s contribution to model output (**Figure 5A**), and the mean absolute SHAP values ranked Age, M_stage, Grade, N_stage, Tumor_Size, Radiation, Liver_metastasis, Marital_status, Chemotherapy, Bone_metastasis, and Brain_metastasis in descending order (**Figure 5B**). Age had the largest mean absolute SHAP value (0.0706; 29.0% of the total contribution), followed by M_stage (0.0399; 16.4%) and Grade (0.0277; 11.4%), whereas Bone_metastasis and Brain_metastasis made smaller individual contributions (**Figure 5C**). **Figure 5D** illustrates how the included variables jointly shifted the model output for a representative patient. These results largely confirmed established clinicopathological prognostic factors and helped explain how the fitted model combined them at the individual-prediction level.

### 3.7 Temporal validation in the 2019–2021 SEER cohort

**Figure 6:**
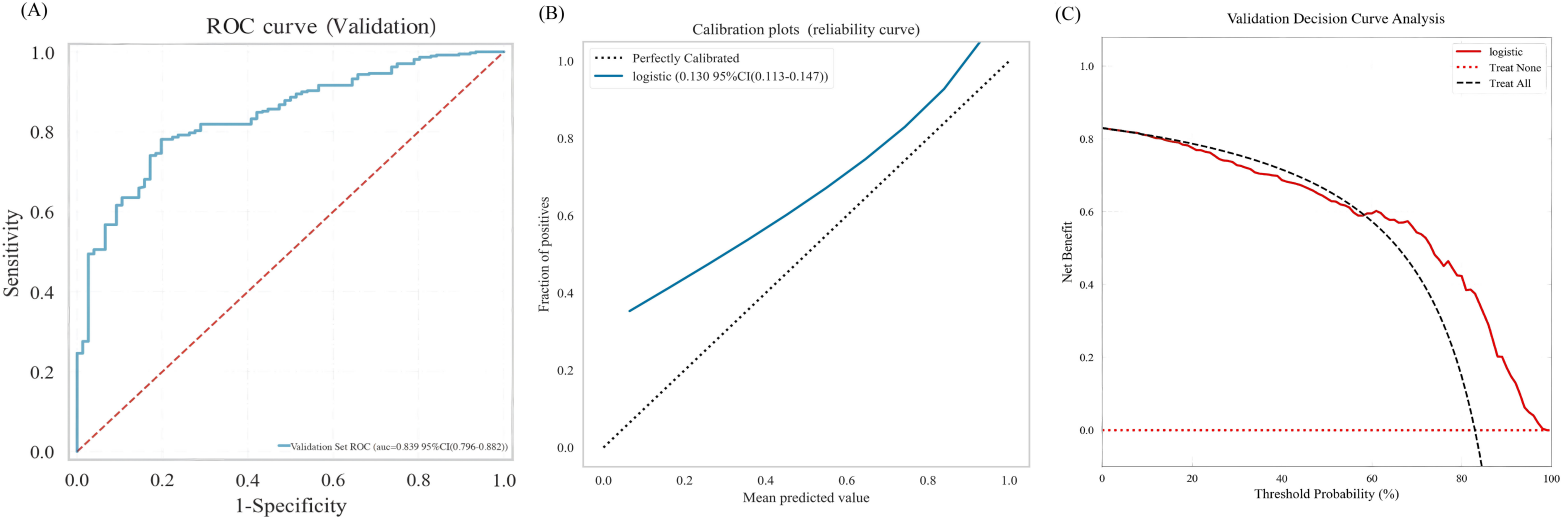
Performance evaluation of the LR model in the 2019–2021 temporal SEER validation cohort. **(A)** ROC curve and corresponding AUC. **(B)** Calibration curve, with the dotted diagonal representing perfect calibration. **(C)** Decision-curve comparison of the model with the treat-all and treat-none strategies across the evaluated threshold probabilities.

In the temporal SEER validation cohort, LR yielded an AUC of 0.839 (95% CI: 0.796–0.882) (**Figure 6A**) and a Brier score of 0.130 (95% CI: 0.113–0.147) (**Figure 6B**). DCA suggested potential net benefit over the treat-all and treat-none strategies across selected threshold probabilities (**Figure 6C**). Because this cohort originated from the same registry as the development cohort, these results indicate temporal performance within SEER but do not establish independent geographic transportability.

### 3.8 Preliminary independent single-center evaluation

**Figure 7:**
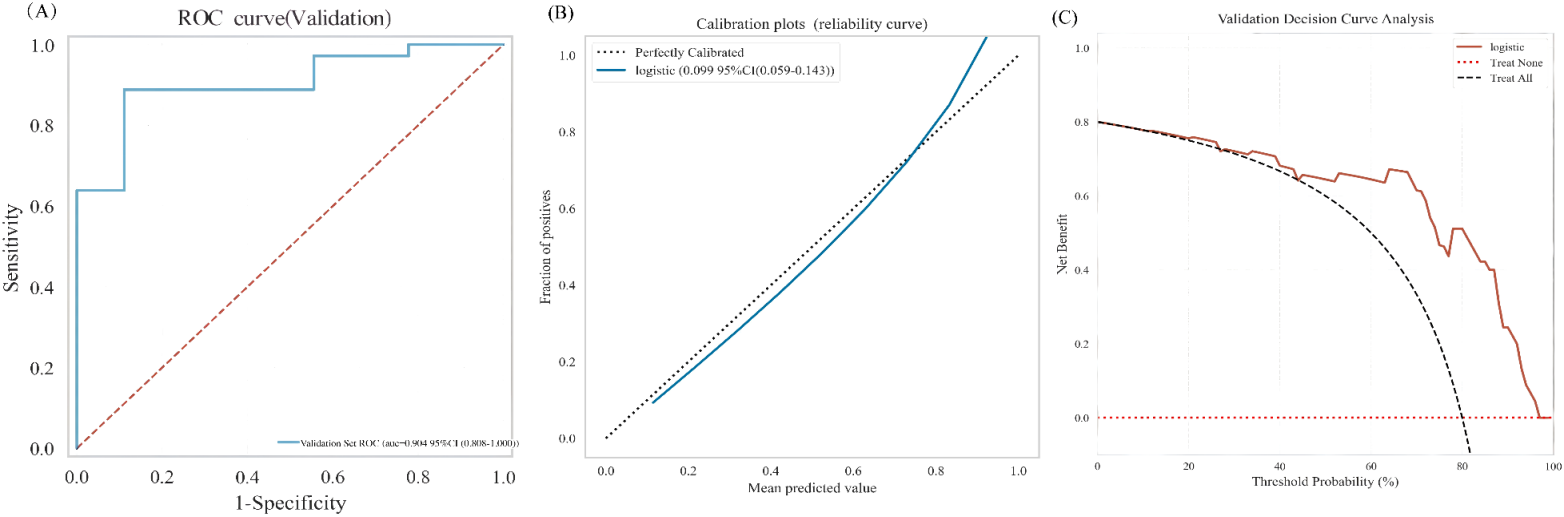
Performance evaluation of the LR model in the preliminary independent single-center cohort from the General Hospital of Ningxia Medical University (2015–2024). **(A)** ROC curve and corresponding AUC. **(B)** Calibration curve, with the dotted diagonal representing perfect calibration. **(C)** Decision-curve comparison of the model with the treat-all and treat-none strategies across the evaluated threshold probabilities.

In the independent single-center cohort, LR yielded an AUC of 0.904 (95% CI: 0.808–1.000) (**Figure 7A**) and a Brier score of 0.099 (95% CI: 0.059–0.143) (**Figure 7B**). DCA suggested potential net benefit across selected threshold probabilities (**Figure 7C**). However, the small sample size (n=45) and wide confidence interval limit the precision of this evaluation. The findings therefore represent preliminary single-center evaluation and do not establish geographic transportability, which requires validation across multiple independent institutions.

Numerical calibration estimates for the three evaluation cohorts are summarized in (**Supplementary Table 7**). The calibration intercept and slope were 0.036 (95% CI: −0.279 to 0.350) and 1.027 (95% CI: 0.793–1.262), respectively, in the held-out internal test cohort; −1.006 (95% CI: −1.296 to −0.716) and 0.979 (95% CI: 0.752–1.205), respectively, in the temporal SEER validation cohort; and −0.382 (95% CI: −1.406 to 0.641) and 1.656 (95% CI: 0.628–2.684), respectively, in the independent single-center cohort.

## 4 Discussion

Atypical pulmonary carcinoid (AC) is characterized by substantial heterogeneity in clinical behavior, while AC-specific clinical practice and prognostic assessment remain incompletely standardized (25). Current morphology-based criteria may not fully distinguish tumors with more aggressive behavior (26), and atypical imaging presentations can complicate diagnosis and TNM staging (27). These limitations support the development of quantitative models that integrate routinely collected clinicopathological information. Such models should complement, rather than replace, pathological assessment, anatomical staging, and multidisciplinary clinical judgment.

The present study developed and evaluated an interpretable model for estimating 3-year all-cause mortality in patients with AC through systematic comparison of seven candidate classifiers. Previous systematic evidence indicates that greater algorithmic complexity does not necessarily translate into better clinical prediction performance (28). In the present analysis, boosting algorithms achieved very high apparent training discrimination but showed a larger decline in held-out data, a pattern consistent with overfitting, whereas LR provided a more balanced combination of held-out discrimination, overall predictive performance, and interpretability. Decision curve analysis suggested potential net benefit across selected threshold probabilities. SHAP quantified the contribution of individual predictors to the fitted output, consistent with its use for transparent explanation in other clinical prediction studies (29). The principal contribution of this study is therefore comparative model evaluation, AC-specific fixed-horizon outcome estimation, temporal evaluation, preliminary single-center assessment, and transparent interpretation rather than algorithmic novelty.

Age had the largest mean absolute SHAP value, indicating that the Age variable contributed substantially to predictions made by the fitted model. The median Age of 65 years in this cohort was consistent with a previous AC prognostic study (10), and older Age has been associated with adverse outcomes in a national cohort of surgically managed atypical pulmonary carcinoid tumors (30). Age may capture differences in physiological reserve, comorbidity burden, treatment tolerance, and competing mortality risk that were not fully available in SEER. These factors provide plausible context for its predictive contribution, although SHAP reflects the fitted association and does not establish a causal mechanism or treatment implication.

Grade and Tumor_Size were also important contributors to model predictions. In the descriptive baseline comparison, patients who died within 3 years had a less favorable Grade distribution and a larger median Tumor_Size than those who survived for at least 3 years. Higher Grade may reflect increasing cellular atypia, proliferative activity, and invasive potential, and its association with prognosis is consistent with previous surgical evidence in atypical pulmonary carcinoid tumors (30). Larger Tumor_Size represents greater tumor burden and may provide more opportunity for regional extension or vascular and lymphatic dissemination; Tumor_Size has also been incorporated into previous prognostic nomograms for lung carcinoid tumors (31). These clinicopathological interpretations provide context for their predictive contribution, but the present retrospective analysis cannot establish the specific biological pathways through which they influence mortality.

Metastatic burden remained an important component of model prediction. M_stage captures the overall presence of distant disease, whereas Bone_metastasis, Brain_metastasis, and Liver_metastasis describe its anatomical distribution. Patients with metastatic lung carcinoid tumors are clinically heterogeneous in disease pattern, treatment, and outcome (32), and organ-specific subdivision of the M component has been proposed for metastatic pulmonary neuroendocrine tumors (33). Clinicopathological series and reviews have also linked nodal involvement and histological characteristics to prognosis (34–36), while population-based prognostic models have incorporated nodal and metastatic characteristics (37). Brain, liver, and bone metastases may impose different clinical burdens through neurological compromise, hepatic dysfunction, skeletal complications, symptom burden, and treatment constraints, although these mechanisms were not directly evaluated. Pairwise correlations indicated overlap among metastasis-related predictors, but all variance inflation factors were below 2.50. Excluding M_stage or the three organ-specific variables produced only modest numerical changes in cross-validated and temporal-validation AUCs. The full set was retained because it captures related but non-identical aspects of metastatic status and distribution; the descriptive sensitivity analysis supports stability but not causal independence or the necessity of every predictor (**Supplementary Table 5**).

Treatment variables require cautious interpretation. Multicenter clinical evidence indicates that management of atypical pulmonary carcinoid depends on stage, histology, operability, patient condition, and institutional practice (11). Surgery remains central for appropriately selected patients with localized pulmonary carcinoid tumors (38), whereas evidence for radiotherapy and systemic treatment is heterogeneous and largely retrospective (39). The contributions of Chemotherapy and Radiation to prediction likely reflect confounding by indication, baseline disease severity, and non-random treatment selection, because these treatments are preferentially used in specific high-risk clinical settings. Detailed regimens, doses, treatment intent, response, and performance status were unavailable. Accordingly, these variables represent prognostic context rather than treatment-effect estimates and should not guide treatment selection.

In addition to conventional clinicopathological characteristics, Marital_status was retained as a predictor. It may proxy differences in social support, caregiver availability, healthcare access, treatment adherence, timely presentation, and continuity of follow-up. These mechanisms were not directly measured, and the meaning of marital categories may vary across cultures and healthcare systems. Its contribution is therefore hypothesis-generating and should be evaluated alongside direct measures of social support and socioeconomic vulnerability in future cohorts.

Previous pulmonary carcinoid prognostic tools have primarily used regression-based approaches. A SEER-based model examined the role of surgery in atypical bronchopulmonary carcinoid tumors (7), and another SEER study with external validation developed an AC-specific prognostic nomogram (10). Other nomograms have addressed long-term cancer-specific survival in lung carcinoid tumors (31) and resected non-small-cell pulmonary neuroendocrine tumors (37). Population-based analyses have also characterized incidence and survival across lung neuroendocrine neoplasms (40). The Rachel score incorporated clinical and pathological characteristics, including Ki-67, for overall and progression-free survival (41). Consistent with these tools, the present model identified Age, Grade, nodal stage, metastatic stage, and organ-specific metastases as important predictors. Its distinguishing features are the AC-specific fixed 3-year all-cause mortality endpoint, comparison of seven classifiers, temporal evaluation, preliminary single-center assessment, and SHAP-based interpretation. However, no published nomogram or the Rachel score was reconstructed or directly applied to the present cohorts. The benchmark analysis compared only internally constructed TNM and parsimonious clinical specifications; therefore, it cannot establish superiority over published models. Direct head-to-head comparison in independent multi-institutional cohorts is required.

The selected predictors are generally obtainable in routine practice and could support development of an electronic or web-based calculator after independent validation. A future tool might present a standardized 3-year mortality estimate alongside TNM stage, histology, performance status, comorbidities, and treatment considerations to support multidisciplinary discussion and prospective risk-stratification research. However, the present study established neither a clinically validated decision threshold nor a surveillance or treatment pathway, and no impact study has shown improved patient outcomes. Geographic transportability must first be demonstrated across multiple independent institutions and healthcare settings; the model is not ready for clinical implementation.

### 4.1 Limitations

This study has several limitations. First, the retrospective registry and single-center data may be affected by selection bias, information bias, residual confounding, and non-random treatment allocation; treatment-related predictors are predictive rather than causal. Second, clinically relevant variables such as Ki-67, molecular biomarkers, performance status, comorbidities, and detailed treatment information were unavailable or incompletely recorded. Third, multiple imputation was performed on the complete 2000–2021 SEER dataset before separation of the development, internal-test, and temporal cohorts. Consequently, observed information from evaluation cohorts contributed to estimation of the imputation models used for the development data. This violates strict separation of preprocessing, may reduce apparent differences between cohorts, and could yield optimistic estimates of discrimination and calibration. Restricting feature selection and model fitting to the training set does not eliminate this leakage, and its magnitude cannot be quantified without re-analysis. A rigorous future workflow should fit imputation within each training fold and maintain independent preprocessing of temporal or external cohorts. Finally, the temporal cohort came from the same registry, and the independent cohort included only 45 patients from one center. Geographic transportability therefore remains unestablished and requires prospective validation across multiple independent institutions and healthcare settings.

## 5 Conclusion

In summary, systematic comparison of seven candidate algorithms identified LR as the most consistent and interpretable approach for estimating 3-year all-cause mortality in patients with AC. The model showed stable discrimination in internal and same-registry temporal evaluation and promising but imprecise results in one small single-center cohort. These findings do not establish geographic transportability or readiness for clinical implementation. Independent multi-institutional validation, fully separated preprocessing, clinically meaningful thresholds, and clinical-impact assessment are required before use in practice.

## Data Availability

Publicly available datasets were analyzed in this study. The SEER data are available from the National Cancer Institute at https://seer.cancer.gov/. The de-identified single-center data from the General Hospital of Ningxia Medical University are not publicly available because of institutional and patient privacy restrictions. Further inquiries may be directed to the corresponding author.
