# Supplementary material for: A dual-validated machine learning model for predicting 3-year mortality in atypical pulmonary carcinoid, a rare neuroendocrine tumor
Source: Front Endocrinol (Lausanne). 2026 Aug 3;17:1901646. doi: 10.3389/fendo.2026.1901646 (PMC13477948; doi:10.3389/fendo.2026.1901646)
Supplement: Supplementary file 1 [file SupplementaryFile1.docx]

**TRIPOD+AI Checklist and Manuscript Mapping**

This completed checklist maps the applicable TRIPOD+AI reporting items to the final clean revised manuscript, “A dual-validated machine learning model for predicting 3-year mortality in atypical pulmonary carcinoid, a rare neuroendocrine tumor.” Section, table, figure, and supplementary-table references correspond to the final clean revised manuscript and the final revised supplementary materials. Items that were not applicable to this retrospective prediction-model study are identified as such, and partial reporting is stated explicitly where formal fairness analysis, complete model coefficients, or full primary-analysis code were not available.

| **Item** | **Topic** | **How addressed** | **Manuscript location** | **Status** |
| --- | --- | --- | --- | --- |
| 1 | Title | The title identifies the machine-learning prediction model, target population (atypical pulmonary carcinoid), and 3-year mortality outcome. | Title | Reported |
| 2 | Abstract | The structured abstract reports the objective, data sources, cohort roles, predictors, candidate models, discrimination, imprecision of the small single-center cohort, unestablished geographic transportability, and key next steps. | Abstract | Reported |
| 3a | Background/existing models | The healthcare context, prognostic uncertainty, TNM staging, and existing pulmonary carcinoid prognostic models are described. | Introduction, paragraphs 1–3 | Reported |
| 3b | Target population/intended purpose/users | The AC-specific population and fixed-horizon risk-estimation purpose are stated. Potential future use is discussed only after independent multi-institutional validation, threshold definition, and impact assessment. | Introduction, final paragraph; Discussion, implementation paragraph | Reported |
| 3c | Known health inequalities | Marital_status is discussed as a proxy for social support, healthcare access, treatment adherence, and socioeconomic vulnerability, including possible cross-cultural variation. No formal health-inequality or fairness analysis was performed. | Discussion, Marital_status paragraph | Context discussed; no formal inequality analysis |
| 4 | Objectives | The development and evaluation objectives are specified in the abstract and at the end of the Introduction. | Abstract (Objectives); Introduction, final paragraph | Reported |
| 5a | Data sources | SEER development/temporal cohorts and the independent single-center cohort are described separately. | Patients and Methods, Section 2.1.1 | Reported |
| 5b | Dates/follow-up | Accrual periods, cohort dates, and the fixed 36-month prediction horizon are reported. | Abstract (Methods); Patients and Methods, Sections 2.1.1 and 2.2.1 | Reported |
| 6a | Setting/centres | The population-based registry setting and the single participating hospital are identified. | Patients and Methods, Section 2.1.1 | Reported |
| 6b | Eligibility criteria | Inclusion and exclusion criteria for SEER and corresponding screening of the single-center cohort are described. | Patients and Methods, Section 2.1.2; Figure 1 | Reported |
| 6c | Treatments | Radiation and Chemotherapy are defined as candidate predictors; their non-random allocation and confounding by indication are explicitly discussed. | Patients and Methods, Section 2.2.1; Discussion, treatment-variables paragraph | Reported |
| 7 | Data preparation | Predictor encoding, missingness assessment, five SPSS multiple imputations, fully conditional specification, export of the completed analysis file, and timing relative to cohort partitioning are described. The resulting cross-cohort information leakage and potential optimism are explicitly explained. | Patients and Methods, Section 2.2.2; Limitations; Supplementary Tables S3–S4 | Reported with explicit limitation |
| 8a | Outcome | All-cause death within 36 months is defined, including event/non-event coding and ascertainment in SEER and the hospital cohort. | Patients and Methods, Sections 2.1.1 and 2.2.1 | Reported |
| 8b | Subjective outcome assessment | Not applicable because mortality status and survival time did not require subjective interpretation. | N/A | Not applicable |
| 8c | Blinding of outcome assessment | Outcome ascertainment was based on registry vital status/survival time and hospital records/follow-up; model-assessor blinding was not relevant to this retrospective objective outcome. | Patients and Methods, Section 2.1.1 | Not applicable |
| 9a | Choice of predictors | The initial candidate variables and training-set-only selection using LASSO and Boruta are described. | Patients and Methods, Sections 2.2.1 and 2.2.3; Results, Section 3.3; Figure 2 | Reported |
| 9b | Predictor definitions | Predictor names, coding categories, and variable types are described in the manuscript and supplementary tables. | Patients and Methods, Sections 2.2.1–2.2.3; Tables 1–2; Supplementary Table S3 | Reported |
| 9c | Subjective predictor assessment | Most predictors were registry-coded or routinely recorded clinical variables; no predictor required a study-specific subjective assessor rating. | N/A | Not applicable |
| 10 | Sample size | The sample was fixed by eligible cases in the prespecified study periods; no formal a priori calculation was performed and all eligible patients were included. | Patients and Methods, Section 2.1.1 | Reported |
| 11 | Missing data | The extent and range of missingness, imputation algorithm, number of imputations, completed analysis dataset, timing before cohort separation, and potential influence on optimistic performance are reported. | Patients and Methods, Section 2.2.2; Limitations; Supplementary Tables S3–S4 | Reported with methodological limitation |
| 12a | Use/partition of data | The development, training, internal test, temporal validation, and single-center evaluation roles are described; feature selection was restricted to the training set after the 7:3 split. | Patients and Methods, Sections 2.1.1 and 2.2.3 | Reported |
| 12b | Predictor handling | Numerical encoding according to prespecified categories and the preprocessing workflow are described. | Patients and Methods, Sections 2.2.2–2.2.3; Supplementary Table S4 | Reported |
| 12c | Model type/building/tuning/internal validation | Seven classifiers, training-set-only feature selection, 10-fold cross-validation, held-out internal testing, and the reviewer-requested post hoc logistic-regression analyses are described. Available and verifiable implementation details are summarized in the supplement. | Patients and Methods, Sections 2.2.3–2.2.4; Supplementary Table S4 | Reported for available materials |
| 12d | Cluster heterogeneity | No clustered or multilevel data structure was modeled and no cluster-specific heterogeneity analysis was performed. | N/A | Not applicable |
| 12e | Performance measures | Discrimination, graphical calibration, calibration intercept, calibration slope, Brier score, precision-recall performance, decision curve analysis, and learning curves are described. Numerical calibration estimates are reported with 95% confidence intervals for all evaluation cohorts. | Patients and Methods, Sections 2.2.3–2.2.4; Results, Sections 3.4–3.8; Supplementary Tables S4 and S7; Figures 3–7 | Reported |
| 12f | Model updating | No recalibration or model updating was performed. | N/A | Not applicable |
| 12g | Prediction calculation in evaluation | The predictor set, selected model type, cohort-specific evaluation procedures, and supplementary logistic-regression workflow are reported. Exact final-model coefficients and intercept were not provided; available post hoc scripts are obtainable on reasonable request. | Patients and Methods, Sections 2.2.3–2.2.4; Results, Sections 3.4–3.8; Supplementary Table S4; Data Sharing Statement | Partially reported |
| 13 | Class imbalance | Class-imbalance handling for the original primary model-development workflow was not fully documented in the retained analysis records. The manuscript and supplementary materials do not report an additional class-balancing procedure for the reviewer-requested post hoc analyses. | Not reported | Not fully documented |
| 14 | Fairness | No formal fairness or subgroup-performance analysis was performed. The potential social and cross-cultural implications of Marital_status are discussed. | Discussion, Marital_status paragraph | Not formally assessed |
| 15 | Model output/threshold | Model performance is probability-based and DCA examines ranges of threshold probabilities. No single clinically validated operational threshold, surveillance pathway, or treatment recommendation was established. | Patients and Methods, Sections 2.2.3–2.2.4; Results, Sections 3.4–3.8; Figures 3 and 6–7; Discussion, implementation paragraph | Reported for exploratory use; no clinical threshold |
| 16 | Development vs evaluation differences | The same-registry temporal cohort and the small single-center cohort are distinguished. The manuscript explicitly states that neither establishes geographic transportability and that multi-institutional validation is required. | Patients and Methods, Section 2.1.1; Results, Sections 3.7–3.8; Discussion; Limitations; Conclusion | Reported |
| 17 | Ethics | Ethics approval, approval number, and waiver of informed consent for retrospective anonymized records are reported. | Patients and Methods, Section 2.1.1; Ethics Approval and Informed Consent | Reported |
| 18a | Funding | Funding sources and grant numbers are listed. The role of the funders was not specified in the manuscript. | Funding | Partially reported; funder role not stated |
| 18b | Conflicts | A conflict-of-interest statement is provided. | Disclosure | Reported |
| 18c | Protocol | The manuscript states that no formal study protocol was prospectively published. | Statement immediately following the Data Sharing Statement | Reported |
| 18d | Registration | The manuscript states that the study was not registered. | Statement immediately following the Data Sharing Statement | Reported |
| 18e | Data sharing | SEER availability and restrictions governing de-identified single-center data are described. | Data Sharing Statement | Reported |
| 18f | Code sharing | The script for the reviewer-requested post hoc sensitivity and benchmark analyses is available from the corresponding author on reasonable request. Full code for the original primary model-development workflow is not stated to be available. | Data Sharing Statement; Supplementary Table S4 | Partially reported; post hoc code available on request |
| 19 | Patient/public involvement | The manuscript states that patients and the public were not involved in study design, conduct, reporting, or dissemination planning. | Statement immediately following the Data Sharing Statement | Reported |
| 20a | Participant flow | Cohort numbers and allocation are reported in the abstract and Methods, and participant selection is shown in the flow diagram. | Abstract; Patients and Methods, Sections 2.1.1–2.1.2; Figure 1 | Reported |
| 20b | Characteristics/events/missingness | Baseline characteristics, development-cohort outcome-event counts, training/test comparisons, and variable-specific missingness are reported. | Results, Sections 3.1–3.2; Tables 1–2; Supplementary Table S3 | Reported |
| 20c | Comparison of development/evaluation data | The development training and internal test sets are compared in Table 2. No formal baseline comparison between the development and external cohorts was performed; cohort sources, roles, and limitations are described. | Table 2; Patients and Methods, Section 2.1.1; Results, Sections 3.7–3.8; Limitations | Partially reported; no formal external-cohort baseline comparison |
| 21 | Participants/events in analyses | Sample sizes are reported for all main cohorts. Outcome-event counts are reported for the development analyses and for the internal-test, temporal-validation, and single-center cohorts in the supplementary calibration table. | Abstract; Patients and Methods; Results, Sections 3.1–3.8; Tables 1–2; Supplementary Tables S5–S7 | Reported |
| 22 | Full model specification | The final predictor set, model type, and evaluation procedures are reported. A complete coefficient equation with intercept was not provided; supplementary logistic-regression procedures are documented and the available post hoc script is provided on reasonable request. | Patients and Methods, Sections 2.2.3–2.2.4; Results, Sections 3.3–3.8; Supplementary Table S4; Data Sharing Statement | Partially reported; full equation unavailable |
| 23a | Performance with CIs | Primary AUCs with 95% confidence intervals, Brier scores, and calibration intercepts and slopes with 95% confidence intervals are reported across the evaluated cohorts; supplementary sensitivity and benchmark AUCs are descriptive. | Results, Sections 3.4–3.8; Figures 3–7; Supplementary Tables S1–S2 and S5–S7 | Reported |
| 23b | Cluster heterogeneity | No cluster-specific performance or heterogeneity analysis was performed. | N/A | Not applicable |
| 24 | Model updating | No model updating was performed. | N/A | Not applicable |
| 25 | Interpretation/fairness | Results are interpreted in relation to prior studies, model complexity, predictor meaning, treatment confounding, internally constructed benchmark models, and intended use. The Discussion explicitly states that published nomograms were not directly compared and that geographic transportability is unestablished. | Discussion | Interpretation reported; fairness not formally assessed |
| 26 | Limitations | Retrospective design, bias and confounding, unavailable predictors, cross-cohort imputation leakage and potential optimism, same-registry temporal evaluation, and the small single-center evaluation are discussed. | Limitations | Reported |
| 27a | Poor/unavailable input data | No operational prediction tool was implemented, and procedures for missing or unavailable inputs at the point of use were therefore not established. | Discussion, implementation paragraph; Limitations | Not applicable at current exploratory stage |
| 27b | User interaction/expertise | No user-facing implementation tool exists; required user interaction and expertise have therefore not been specified. | Discussion, implementation paragraph | Not applicable at current exploratory stage |
| 27c | Next steps | Independent multi-institutional validation, fully separated preprocessing, clinically meaningful thresholds, and clinical-impact assessment are identified as next steps. | Discussion; Limitations; Conclusion | Reported |
