# Supplementary material for: A dual-validated machine learning model for predicting 3-year mortality in atypical pulmonary carcinoid, a rare neuroendocrine tumor
Source: Front Endocrinol (Lausanne). 2026 Aug 3;17:1901646. doi: 10.3389/fendo.2026.1901646 (PMC13477948; doi:10.3389/fendo.2026.1901646)
Supplement: Supplementary file 2 [file Table1.docx]

**Supplementary Table S1. Apparent performance of the seven classifiers in the training set**

| **ML model** | **AUC (95% CI)** | **Cutoff (95% CI)** | **Accuracy (95% CI)** | **Sensitivity (95% CI)** | **Specificity (95% CI)** | **PPV (95% CI)** | **NPV (95% CI)** | **F1 score (95% CI)** | **Kappa (95% CI)** |
| --- | --- | --- | --- | --- | --- | --- | --- | --- | --- |
| LR | 0.811 (0.778-0.845) | 0.715(0.693-0.737) | 0.769(0.755-0.782) | 0.797(0.767-0.827) | 0.695(0.662-0.727) | 0.871(0.864-0.879) | 0.573(0.544-0.601) | 0.832(0.819-0.845) | 0.462(0.445-0.478) |
| XGBoost | 0.997 (0.995-1.000) | 0.657(0.642-0.672) | 0.983(0.979-0.988) | 0.987(0.984-0.991) | 0.972(0.963-0.982) | 0.989(0.986-0.993) | 0.967(0.958-0.977) | 0.988(0.985-0.991) | 0.958(0.947-0.969) |
| LightGBM | 0.995 (0.992-0.998) | 0.631(0.599-0.663) | 0.969(0.965-0.973) | 0.969(0.962-0.976) | 0.97(0.959-0.981) | 0.988(0.984-0.992) | 0.924(0.908-0.940) | 0.978(0.976-0.981) | 0.925(0.915-0.934) |
| AdaBoost | 0.826 (0.795-0.857) | 0.567(0.561-0.574) | 0.779(0.762-0.797) | 0.802(0.763-0.841) | 0.721(0.683-0.759) | 0.882(0.872-0.891) | 0.589(0.554-0.623) | 0.839(0.822-0.857) | 0.489(0.468-0.509) |
| GBDT | 0.914 (0.892-0.937) | 0.692(0.637-0.747) | 0.857(0.838-0.877) | 0.885(0.842-0.928) | 0.785(0.737-0.834) | 0.915(0.900-0.930) | 0.734(0.674-0.795) | 0.899(0.882-0.916) | 0.655(0.623-0.687) |
| KNN | 0.866 (0.842-0.890) | 0.8(0.800-0.800) | 0.783(0.778-0.788) | 0.794(0.782-0.806) | 0.756(0.733-0.779) | 0.894(0.886-0.902) | 0.587(0.578-0.596) | 0.841(0.836-0.846) | 0.505(0.496-0.515) |
| SVM | 0.820 (0.784-0.856) | 0.821(0.815-0.826) | 0.798(0.793-0.803) | 0.818(0.804-0.831) | 0.747(0.730-0.765) | 0.893(0.888-0.898) | 0.614(0.601-0.626) | 0.854(0.848-0.859) | 0.53(0.523-0.536) |

**Abbreviations:** AUC, area under the receiver operating characteristic curve; CI, confidence interval; GBDT, gradient boosting decision tree; KNN, k-nearest neighbors; LightGBM, light gradient boosting machine; LR, logistic regression; NPV, negative predictive value; PPV, positive predictive value; SVM, support vector machine; XGBoost, extreme gradient boosting.

**Supplementary Table S2. Initial seven-classifier comparison in the held-out internal test set**

| **ML model** | **AUC (95% CI)** | **Cutoff (95% CI)** | **Accuracy (95% CI)** | **Sensitivity (95% CI)** | **Specificity (95% CI)** | **PPV (95% CI)** | **NPV (95% CI)** | **F1 score (95% CI)** | **Kappa (95% CI)** |
| --- | --- | --- | --- | --- | --- | --- | --- | --- | --- |
| LR | 0.800 (0.732–0.869) | 0.715(0.693-0.737) | 0.753(0.716-0.790) | 0.793(0.729-0.857) | 0.648(0.570-0.727) | 0.855(0.833-0.876) | 0.558(0.498-0.618) | 0.821(0.788-0.855) | 0.42(0.355-0.484) |
| XGBoost | 0.732 (0.653-0.811) | 0.657(0.642-0.672) | 0.73(0.718-0.742) | 0.801(0.778-0.825) | 0.545(0.474-0.615) | 0.821(0.801-0.840) | 0.514(0.494-0.534) | 0.81(0.802-0.819) | 0.339(0.296-0.381) |
| LightGBM | 0.747 (0.669-0.825) | 0.631(0.599-0.663) | 0.742(0.732-0.753) | 0.808(0.791-0.825) | 0.572(0.525-0.619) | 0.831(0.817-0.844) | 0.536(0.518-0.553) | 0.819(0.811-0.827) | 0.372(0.341-0.403) |
| AdaBoost | 0.799 (0.730-0.868) | 0.567(0.561-0.574) | 0.751(0.729-0.773) | 0.784(0.744-0.824) | 0.666(0.622-0.709) | 0.859(0.847-0.871) | 0.547(0.511-0.583) | 0.819(0.798-0.840) | 0.421(0.386-0.456) |
| GBDT | 0.798 (0.729-0.867) | 0.692(0.637-0.747) | 0.774(0.754-0.794) | 0.821(0.794-0.848) | 0.652(0.595-0.709) | 0.86(0.841-0.878) | 0.587(0.548-0.625) | 0.84(0.824-0.855) | 0.457(0.407-0.507) |
| KNN | 0.710 (0.627-0.793) | 0.8(0.800-0.800) | 0.7(0.671-0.729) | 0.737(0.684-0.791) | 0.603(0.547-0.660) | 0.828(0.814-0.843) | 0.476(0.433-0.518) | 0.779(0.751-0.807) | 0.315(0.272-0.358) |
| SVM | 0.728 (0.641-0.816) | 0.821(0.815-0.826) | 0.713(0.689-0.738) | 0.727(0.690-0.763) | 0.679(0.650-0.709) | 0.854(0.843-0.865) | 0.492(0.460-0.524) | 0.785(0.762-0.808) | 0.364(0.323-0.405) |

**Abbreviations:** AUC, area under the receiver operating characteristic curve; CI, confidence interval; GBDT, gradient boosting decision tree; KNN, k-nearest neighbors; LightGBM, light gradient boosting machine; LR, logistic regression; NPV, negative predictive value; PPV, positive predictive value; SVM, support vector machine; XGBoost, extreme gradient boosting.

## Supplementary Table S3. Missing-data profile and multiple-imputation details for candidate predictors

| **Predictor** | **Missing, n** | **Missing, %** | **Variable type** | **Imputation approach** |
| --- | --- | --- | --- | --- |
| Primary_Site | 137 | 7.8 | Categorical | Automatic FCS; logistic-type regression |
| Grade | 919 | 52.6 | Categorical | Automatic FCS; logistic-type regression |
| Laterality | 47 | 2.7 | Binary categorical | Automatic FCS; logistic-type regression |
| T_stage | 697 | 39.9 | Categorical | Automatic FCS; logistic-type regression |
| N_stage | 670 | 38.4 | Categorical | Automatic FCS; logistic-type regression |
| M_stage | 627 | 35.9 | Binary categorical | Automatic FCS; logistic-type regression |
| Systemic_Sur_Seq | 204 | 11.7 | Binary categorical | Automatic FCS; logistic-type regression |
| Tumor_Size | 204 | 11.7 | Scale | Automatic FCS; linear regression |
| Bone_metastasis | 373 | 21.4 | Binary categorical | Automatic FCS; logistic-type regression |
| Brain_metastasis | 373 | 21.4 | Binary categorical | Automatic FCS; logistic-type regression |
| Liver_metastasis | 371 | 21.2 | Binary categorical | Automatic FCS; logistic-type regression |
| Lung_metastasis | 377 | 21.6 | Binary categorical | Automatic FCS; logistic-type regression |
| Lymph_node_metastases | 859 | 49.2 | Binary categorical | Automatic FCS; logistic-type regression |
| Marital_status | 67 | 3.8 | Categorical | Automatic FCS; logistic-type regression |
| Sex | 0 | 0.0 | Categorical | Not required |
| Race | 0 | 0.0 | Categorical | Not required |
| Surgery_Radiation_Sequence | 0 | 0.0 | Categorical | Not required |
| Radiation | 0 | 0.0 | Binary categorical | Not required |
| Chemotherapy | 0 | 0.0 | Binary categorical | Not required |
| Rural_Urban | 0 | 0.0 | Categorical | Not required |
| Age | 0 | 0.0 | Scale | Not required |

Note. FCS, fully conditional specification. Missing data were handled using the Automatic multiple-imputation procedure in IBM SPSS Statistics version 25.0, with five imputations specified. For the non-monotone missingness pattern, FCS was used, with linear regression for scale variables and logistic-type regression models for categorical variables. The completed data file available from the original SPSS imputation workflow was exported for subsequent analyses in R and Python. Imputation was performed before temporal cohort separation and the subsequent training-internal-test split.

## Supplementary Table S4. Reproducibility and implementation information

| **Component** | **Reported implementation** |
| --- | --- |
| Outcome coding | All-cause death within 36 months was coded as 1; survival to at least 36 months was coded as 0 for model fitting. |
| Primary cohort partition | The 2000-2018 development cohort was divided 7:3 into training and held-out internal test sets; the 2019-2021 SEER cohort and the single-center cohort were evaluated separately. |
| Missing data | SPSS Automatic multiple imputation with five imputations and FCS for non-monotone missingness; linear regression was used for scale variables and logistic-type regression models for categorical variables. The completed data file available from the original SPSS workflow was exported for R and Python analyses. Imputation preceded cohort separation and the 7:3 split, so preprocessing was not fully isolated across development and evaluation cohorts and may have introduced information sharing and optimistic performance estimates. |
| Feature selection | LASSO regression and Boruta were applied exclusively within the training set after the 7:3 split; 11 predictors were retained. |
| Candidate classifiers | LR, XGBoost, LightGBM, GBDT, AdaBoost, SVM, and KNN. |
| Internal validation | Ten-fold cross-validation was performed within the training set; the held-out internal test set was used for performance assessment. |
| Performance measures | ROC AUC, calibration plots, calibration intercept, calibration slope, Brier score, precision-recall analysis, decision curve analysis, and SHAP interpretation. Calibration intercepts and slopes were estimated separately in each evaluation cohort using logistic recalibration of the observed outcome on the logit-transformed predicted probability; ideal values were 0 and 1, respectively, and Wald 95% confidence intervals were reported. |
| Reviewer-requested post hoc analyses | Unpenalized binomial logistic regression with a logit link; complete 2000-2018 development cohort; stratified 10-fold cross-validation; final fit in the development cohort and evaluation in the 2019-2021 temporal cohort. A fixed seed was used for reproducibility; the complete analysis script is available from the corresponding author upon reasonable request. |
| Post hoc analysis code | The script used for the reviewer-requested sensitivity and benchmark analyses is available from the corresponding author upon reasonable request. |
| Classifier setting information | The manuscript reports the candidate classifiers, data partition, feature-selection procedure, internal validation, and performance measures documented in the retained analysis records. No unverified settings were added. |

## Supplementary Table S5. Multicollinearity assessment and metastatic-variable sensitivity analyses

**A. Pairwise correlations among metastatic predictors**

| **Predictor** | **M_stage** | **Bone_metastasis** | **Brain_metastasis** | **Liver_metastasis** |
| --- | --- | --- | --- | --- |
| M_stage | 1.000 | 0.507 | 0.335 | 0.636 |
| Bone_metastasis | 0.507 | 1.000 | 0.184 | 0.273 |
| Brain_metastasis | 0.335 | 0.184 | 1.000 | 0.130 |
| Liver_metastasis | 0.636 | 0.273 | 0.130 | 1.000 |

**B. Variance inflation factors for the full 11-predictor model**

| **Predictor** | **VIF** |
| --- | --- |
| Grade | 1.08 |
| N_stage | 1.30 |
| M_stage | 2.49 |
| Bone_metastasis | 1.38 |
| Brain_metastasis | 1.19 |
| Liver_metastasis | 1.73 |
| Marital_status | 1.15 |
| Radiation | 1.29 |
| Chemotherapy | 1.41 |
| Age | 1.17 |
| Tumor_Size | 1.17 |

**C. Sensitivity analyses**

| **Model** | **Evaluation** | **Events/N** | **AUC** | **ΔAUC vs full** | **Interpretation** |
| --- | --- | --- | --- | --- | --- |
| Full 11-predictor model | 10-fold CV (out-of-fold) | 363/1,301 | 0.787 | Reference | Post hoc reference specification. |
| Full 11-predictor model | Temporal validation | 76/446 | 0.843 | Reference | Post hoc reference specification. |
| Without M_stage | 10-fold CV (out-of-fold) | 363/1,301 | 0.782 | −0.005 | Performance broadly maintained. |
| Without M_stage | Temporal validation | 76/446 | 0.832 | −0.011 | Performance broadly maintained. |
| M_stage retained; Bone_metastasis, Brain_metastasis, and Liver_metastasis excluded | 10-fold CV (out-of-fold) | 363/1,301 | 0.785 | −0.002 | Performance broadly maintained. |
| M_stage retained; Bone_metastasis, Brain_metastasis, and Liver_metastasis excluded | Temporal validation | 76/446 | 0.838 | −0.005 | Performance broadly maintained. |

Note. These post hoc sensitivity analyses used the same unpenalized logistic-regression procedure for all three predictor specifications. Performance was assessed by stratified 10-fold cross-validation in the complete 2000-2018 development cohort and temporal validation in the 2019-2021 SEER cohort. Comparisons were descriptive; no formal hypothesis test of AUC differences was performed. VIF values below 5 were considered not to indicate problematic multicollinearity.

## Supplementary Table S6. Incremental predictive value compared with simpler benchmark models

| **Model** | **Predictors** | **Evaluation** | **Events/N** | **AUC** | **ΔAUC vs TNM-only** |
| --- | --- | --- | --- | --- | --- |
| TNM-only model | T_stage, N_stage, M_stage | 10-fold CV (out-of-fold) | 363/1,301 | 0.704 | Reference |
| TNM-only model | T_stage, N_stage, M_stage | Temporal validation | 76/446 | 0.775 | Reference |
| Parsimonious clinical model | Age, Grade, Tumor_Size, T_stage, N_stage, M_stage | 10-fold CV (out-of-fold) | 363/1,301 | 0.775 | +0.071 |
| Parsimonious clinical model | Age, Grade, Tumor_Size, T_stage, N_stage, M_stage | Temporal validation | 76/446 | 0.816 | +0.041 |
| Full 11-predictor model | Grade, N_stage, M_stage, Bone_metastasis, Brain_metastasis, Liver_metastasis, Marital_status, Radiation, Chemotherapy, Age, Tumor_Size | 10-fold CV (out-of-fold) | 363/1,301 | 0.787 | +0.083 |
| Full 11-predictor model | Grade, N_stage, M_stage, Bone_metastasis, Brain_metastasis, Liver_metastasis, Marital_status, Radiation, Chemotherapy, Age, Tumor_Size | Temporal validation | 76/446 | 0.843 | +0.068 |

Note. These post hoc benchmark analyses used the same unpenalized logistic-regression procedure for all model specifications. Performance was assessed by stratified 10-fold cross-validation in the complete 2000-2018 development cohort and temporal validation in the 2019-2021 SEER cohort. Comparisons were descriptive and do not establish statistical or clinical superiority over existing prognostic tools.

## Supplementary Table S7. Numerical calibration of the final logistic-regression model

| **Cohort** | **Events/N** | **Calibration intercept (95% CI)** | **Calibration slope (95% CI)** |
| --- | --- | --- | --- |
| Held-out internal test cohort | 113/391 | 0.036 (−0.279 to 0.350) | 1.027 (0.793–1.262) |
| 2019–2021 temporal SEER cohort | 76/446 | −1.006 (−1.296 to −0.716) | 0.979 (0.752–1.205) |
| Independent single-center cohort | 9/45 | −0.382 (−1.406 to 0.641) | 1.656 (0.628–2.684) |

Note. Calibration intercepts and slopes were estimated jointly using logistic recalibration of the observed 3-year mortality outcome on the logit-transformed predicted probability. Ideal values are 0 for the intercept and 1 for the slope. CI, confidence interval.
